# Two Mathematical Models for Generation of Crowned Tooth Surface

**DOI:** 10.1155/2014/641091

**Published:** 2014-01-21

**Authors:** Laszlo Kelemen, Jozsef Szente

**Affiliations:** University of Miskolc, Egyetemvaros, Miskolc 3515, Hungary

## Abstract

Gear couplings are mechanical components to connect shaft ends and eliminate the misalignments. The most important element of the gear coupling is the hub which is an external gear having crowned teeth. The crowned teeth on the hub are typically produced by hobbing. The resulting tooth surface depends on several parameters. It is influenced by the size of the hob and the feed. In this paper two mathematical models of the crowned tooth surface are introduced for the generation of the idealized tooth surfaces. These are the profile-shifting and the two-parameter enveloping methods. Our aim is to compare the obtained crowned tooth profiles for the two examined models and to investigate the results. From our numerical results, it was found that the two profiles show indistinguishable differences.

## 1. Introduction

**Figure 1:**
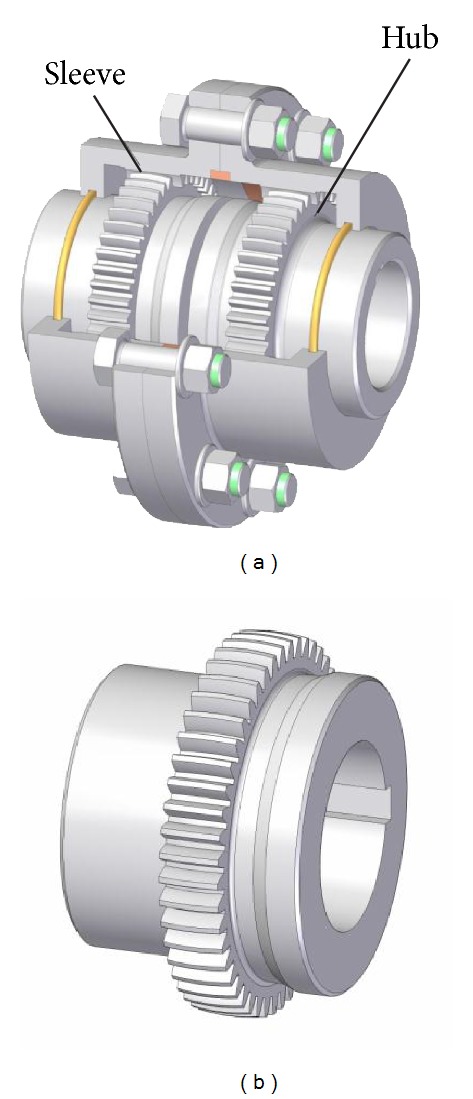
Gear coupling (a) and hub with crowned teeth (b).

Figure 1 shows a gear coupling having two hub-sleeve pairs. The sleeve is an internal gear and the hub is an external gear which has crowned teeth. The two toothed components compose a special gear pair, wherein both numbers of teeth are the same. The gear coupling is able to compensate the misalignment of the coupled shafts by the tooth crowning and backlash. Using a single hub and sleeve, the effect of angular misalignment may be eliminated. In the practice, generally two hub-sleeve pairs are built up as it is shown in Figure 1. In this case, the compensation of the offset misalignment is possible in addition to the angular misalignment.

Gear couplings have been investigated by several researchers. Moked's paper [1] contains a theoretical analysis of toothed coupling including the kinematics of motion, the optimal backlash, the sliding velocity, the power loss, and the contact stress. Renzo et al. [2] gave a discussion of the operation of gear coupling at angular misalignment. They analyzed mesh, tooth bearing, contact cycles and backlash, load distribution, and special tooth forms. Alfares et al. [3] determined the clearance distribution between meshing teeth of misaligned gear coupling. Yi [4] presented an analysis of three-dimensional meshing of crown gear coupling. The authors of this paper derived the tooth surfaces of the hub and sleeve [5] and analyzed the gear meshing of gear coupling [6] using theory of gearing [7].

The most complicated component of the gear coupling is the hub having crowned tooth surfaces (Figure 1), which basically affects the operation of coupling, load carrying capacity, and ability to compensate the misalignments. A typical solution for the production of tooth surfaces is the hobbing. In view of the fact that the resulting surfaces will vary depending on cutter diameter and the motion parameters, hereinafter we create two approximate models which are based on profile shifting [5] and two-parameter enveloping [7, 8].

## 2. Gear Manufacturing of the Crowned Hub

**Figure 2:**
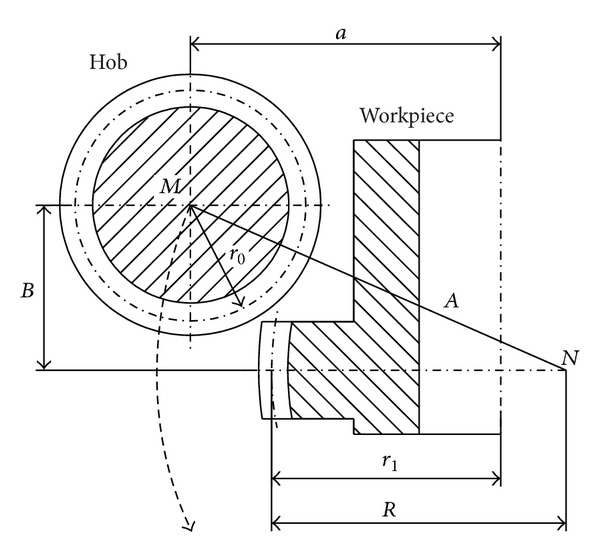
A conceptual sketch for the manufacture of crowned tooth surfaces.

The crowned teeth of the coupling hub can be produced in hobbing by coordinated movement of the workpiece and the tool according to Figure 2.

To produce the crowned tooth surfaces the tool moves along a circular path as it is shown in Figure 2. Earlier machine tools have used special-shaped templates for the necessary relative movement. The modern CNC hobbing machines permit the radial motion of the workpiece-table and the axial movement of the tool.

Reference [5] contains an analysis for the manufacture of the hub teeth. During production the centre distance varies continually. The instantaneous value of centre distance is

(1) $$\begin{array}{c} a=\sqrt{A^{2}-B^{2}}-R+r_{1}, \end{array}$$

where *A* and *B* are the current radial and axial positions of the hob, respectively. *R* is the crowning parameter and *r*_1_ is the radius of pitch circle for the workpiece.

The maximum value of centre distance is

(2) $$\begin{array}{c} a_{\max}=r_{0}+r_{1}, \end{array}$$

where *r*_0_ is the radius of pitch cylinder for the hob.

The radius of circular arc of the relative movement between the tool and the workpiece can be calculated by the sum of the pitch radius of the hob and the crowning parameter:

(3) $$\begin{array}{c} A=r_{0}+R. \end{array}$$

## 3. Mathematical Model for Crowned Tooth Surfaces Using Profile Shifting

Actual shape of the crowned tooth surface depends on the diameter of the hob and the magnitude of tangential, radial, and axial feeds. Accordingly, the real tooth surfaces of the same gear will vary with different values of the said parameters.

To prepare mathematical model for tooth surfaces is needed to determine the load carrying capacity of gear coupling or analyzing the operation. The best solution is a theoretical or ideal surface, which is independent of the mentioned characteristics but gives a good approximation of the real tooth surface.

**Figure 3:**
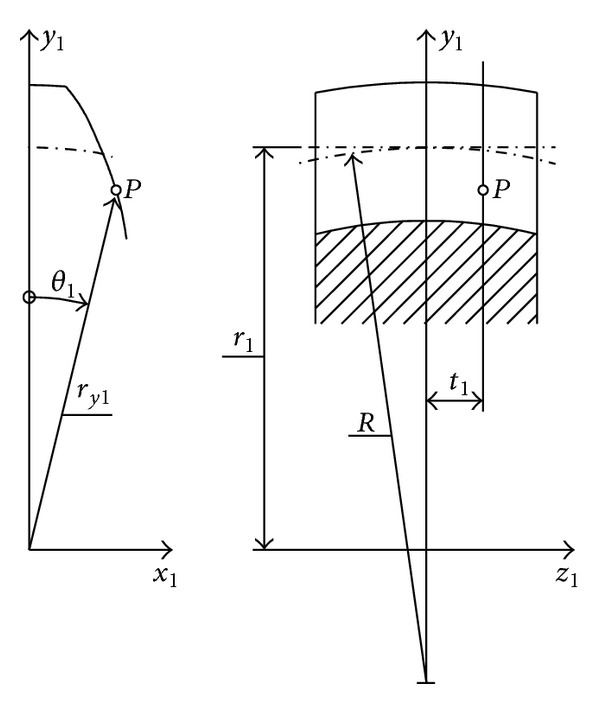
Crowned tooth surface.

The first model for idealized tooth surface of crowned gearing will be derived so that involute tooth surfaces having variable profile shifting in parallel transverse planes are assumed [5] (see Figure 3).

System of equations of the tooth surface can be described as

(4) $$\begin{array}{c} x_{1}=r_{y1}\sin\theta_{1},\\ y_{1}=r_{y1}\cos\theta_{1},\\ z_{1}=t_{1}, \end{array}$$

where *r*_*y*1_ is the arbitrary radius along the tooth profile and *θ*_1_ is the tooth angle. One can calculate *θ*_1_ as follows:

(5) $$\begin{array}{c} \theta_{1}=\frac{s}{2r_{1}}+\text{inv}\;\alpha -\text{inv}\;\alpha_{y1}, \end{array}$$

where *s* is the tooth thickness along the pitch cylinder, *r*_1_ is the pitch radius, *α* is the standard pressure angle, and *α*_*y*1_ is the pressure angle at radius *r*_*y*1_. Angle *α*_*y*1_ can be determined by the following equation:

(6) $$\begin{array}{c} \cos\alpha_{y1}=\frac{r_{b1}}{r_{y1}}. \end{array}$$

Here *r*_*b*1_ denotes the radius of base circle. In (5) the inv stands for the involute function; inv *α* = tan*α* − *α*.

The tooth thickness along the pitch cylinder is expressed by

(7) $$\begin{array}{c} s=s_{0}-2\left(R-\sqrt{R^{2}-z_{1}^{2}}\right)\tan\alpha , \end{array}$$

where *s*_0_ is the tooth thickness in the plane *z*_1_ = 0.

All these indicate that *θ*_1_ depends on the radius *r*_*y*1_ and the coordinate *z*_1_ = *t*_1_; that is, in (4)

(8) $$\begin{array}{c} x_{1}=x_{1}\left(t_{1},r_{y1}\right),\\ y_{1}=y_{1}\left(t_{1},r_{y1}\right). \end{array}$$

## 4. Mathematical Model for Crowned Tooth Surfaces Using Two-Parameter Enveloping

The second model for idealized tooth surface will be prepared by two-parameter enveloping based on the base profile of involute geometry. The theory of two-parameter enveloping has been introduced and applied by Litvin et al. [7, 8].

### 4.1. The Generating Surface

**Figure 4:**
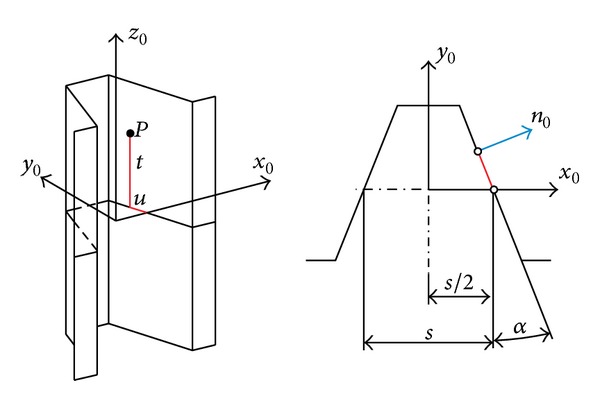
Generating rack.

The generating surfaces are the planes of generating rack obtained from involute base profile (Figure 4). The tooth surface and the surface normal of generating rack are given by the following equations:

(9) $$\begin{array}{c} x_{0}=\frac{s}{2}-u\sin\alpha ,\\ y_{0}=u\cos\alpha ,\\ z_{0}=t, \end{array}$$

(10) $$\begin{array}{c} {n_{x}}_{0}=\cos\alpha ,\\ {n_{y}}_{0}=\sin\alpha ,\\ {n_{z}}_{0}=0. \end{array}$$

In these equations *u* and *t* are the surface parameters, *s* is the tooth thickness measured on the pitch circle in the central plane, and *α* is the standard pressure angle. Interpretation of the notations is shown in Figure 4. The equations are valid for the right plane, but they can easily be prescribed for the left plane on the basis of the symmetry.

### 4.2. Coordinate Systems and the Movement of Members

**Figure 5:**
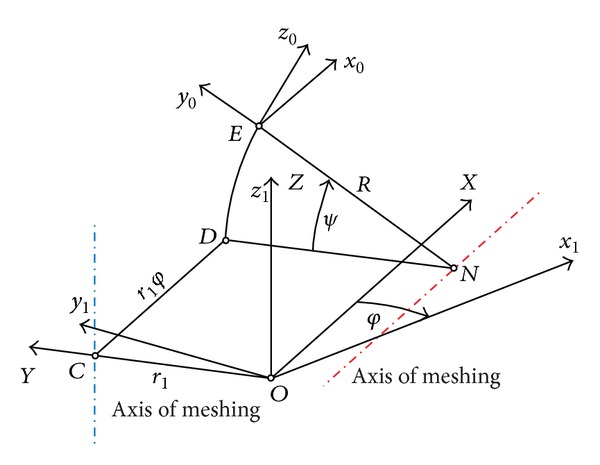
Applied coordinate systems and axes of meshing.

The applied coordinate systems are shown in Figure 5.

The movement of the members is investigated in the stationary coordinate system *S*_*F*_(*O*, *X*, *Y*, *Z*). Coordinate system *S*_1_(*O*, *x*_1_, *y*_1_, *z*_1_) has the same origin as system *S*_*F*_, and axis *z*_1_ coincides with coordinate axis *Z*. Moreover, *S*_1_ is fixed to the hub and it rotates around the axis *Z* with constant angular velocity. The instantaneous rotation of the hub is denoted by angle *φ*.

Coordinate system *S*_0_(*E*, *x*_0_, *y*_0_, *z*_0_) is rigidly connected to the generating rack. It has a screw motion around an axis which is parallel to axis *X* and passing through the point *N*. At a given moment the rotation and translation are characterized by angle *ψ* and distance *r*_1_ *φ*, respectively. In Figure 5, *r*_1_ is the pitch circle radius of the hub and *R* is the crowning parameter.

The relationship between the coordinate systems is given by the following transformation matrices:

(11) $$\begin{array}{c} M_{F0}=\left[\begin{array}{cccc} 1 & 0 & 0 & r_{1}\varphi \\ 0 & \cos\psi & -\sin\psi & r_{1}-R\left(1-\cos\psi \right)\\ 0 & \sin\psi & \cos\psi & R\sin\psi \\ 0 & 0 & 0 & 1 \end{array}\right]\\ M_{1F}=\left[\begin{array}{cccc} \cos\varphi & -\sin\varphi & 0 & 0\\ \sin\varphi & \cos\varphi & 0 & 0\\ 0 & 0 & 1 & 0\\ 0 & 0 & 0 & 1 \end{array}\right], \end{array}$$

where *M*_*F*0_ is the transition matrix from *S*_0_ to *S*_*F*_ and *M*_1*F*_ is the same between *S*_*F*_ and *S*_1_.

### 4.3. Equation of the Crowned Tooth Surface

The crowned tooth surface will be produced by two independent parameters (*φ*, *ψ*). The moving generating surface and its normal are given in the coordinate system *S*_*F*_:

(12) X=r1φ+x0,Y=r1−R(1−cosψ)+y0cosψ−z0sinψ,Z=Rsinψ+y0sinψ+z0cosψ,

(13) Nx=cosα,Ny=sinαcosψ,Nz=sinαsinψ.

System of (12) expresses family of surfaces with four parameters:

(14) $$\begin{array}{c} X=X\left(u,\varphi \right),\\ Y=Y\left(u,t,\psi \right),\\ Z=Z\left(u,t,\psi \right). \end{array}$$

Referring now to the rotating coordinate system *S*_1_, the generating family of surfaces is produced by the following equations:

(15) $$\begin{array}{c} x_{1}=X\cos\varphi -Y\sin\varphi ,\\ y_{1}=X\sin\varphi +Y\cos\varphi ,\\ z_{1}=Z. \end{array}$$

Using (14) and (15) we can determine the relation between the coordinates and parameters:

(16) $$\begin{array}{c} x_{1}=x_{1}\left(u,\varphi ,t,\psi \right),\\ y_{1}=y_{1}\left(u,\varphi ,t,\psi \right),\\ z_{1}=z_{1}\left(u,t,\psi \right). \end{array}$$

System (16) contains four parameters. When one determines the crowned tooth surface, the relationships between the parameters of generating surface *u* and *t* and the parameters of motion *φ* and *ψ* have to be found. More specifically, two additional equations are necessary to define the tooth surface. Formally, these two functions are

(17) $$\begin{array}{c} F_{1}\left(u,\varphi ,t,\psi \right)=0,\\ F_{2}\left(u,\varphi ,t,\psi \right)=0. \end{array}$$

The relationship between the parameters is defined by the axes of meshing (see Figure 5). The axis of meshing is a straight line, which is intersected by the common normal of all contact points of the enveloping and enveloped surfaces.

If *φ* = constant, the relative motion is rotation around the line, which passes through the point *N* and is parallel to the axis *X*. The axis of relative rotational movement coincides with the axis of meshing. Let us denote the position vector of contact point by **R** = *X* **i** + *Y* **j** + *Z* **k**, where **i**, **j**, and **k** are the unit vectors of coordinate directions. The point of contact can be obtained through the axis of meshing and the surface normal. This is expressed by the following equation:

(18) $$\begin{array}{c} R=Ai-\left(R-r_{1}\right)j+BN, \end{array}$$

where *A* denotes the distance from the origin to the point of intersection along the axis of meshing and *B* is the distance from the intersection point to the point of contact measured in normal direction. Converting (18) to scalar equations, one gets

(19) $$\begin{array}{c} X=A+B\cos\alpha ,\\ Y=r_{1}-R+B\sin\alpha \cos\psi ,\\ Z=B\sin\alpha \cos\psi . \end{array}$$

Eliminating *B* from the second and third equations of (19) and substituting *Y* and *Z* from (12), it is concluded that there is a solution if *z*_0_ = 0; that is, the surface parameter becomes zero:

(20) $$\begin{array}{c} t=0. \end{array}$$

(20) corresponds to the first parameter relation, *F*_1_(*u*, *φ*, *t*, *ψ*) = 0. In this case the generating surface has contact points on the base profile in the plane *z*_0_ = 0.

Additional parameter relation can be obtained if *ψ* = constant. Then the relative motion is a rotation around the line which passes through the point *C* and is parallel to the axis *Z*. This line is also an axis of the meshing, so the normal at the contact points intersects it. For any point of contact the connection

(21) $$\begin{array}{c} R=r_{1}j+Gk+HN \end{array}$$

exists, where *G* is the distance along the axis of meshing from the origin to the intersection point and *H* is the distance in normal direction from the intersection point to the contact point. By scalar equations we have

(22) $$\begin{array}{c} X=H\cos\alpha ,\\ Y=r_{1}+H\sin\alpha \cos\psi ,\\ Z=G+H\sin\alpha \cos\psi . \end{array}$$

Expressing *H* from the first equation of (22) and substituting it to the second equation and taking the expressions of *X* and *Y* from (12), we get the following formula:

(23) $$\begin{array}{c} \varphi =\frac{1}{r_{1}\tan\alpha }\left(y_{0}-z_{0}\tan\psi -R\frac{1-\cos\psi }{\cos\psi }\right)-\frac{x_{0}}{r_{1}}. \end{array}$$

Equation (23) satisfies the parameter relation *F*_2_(*u*, *φ*, *t*, *ψ*) = 0 in (17). Since *z*_0_ = 0 from (20), then (23) can be simplified as follows:

(24) $$\begin{array}{c} \varphi =\frac{1}{r_{1}\tan\alpha }\left(y_{0}-R\frac{1-\cos\psi }{\cos\psi }\right)-\frac{x_{0}}{r_{1}}. \end{array}$$

Substituting the expressions from (9) into (24) we obtain

(25) $$\begin{array}{c} \varphi =\frac{1}{r_{1}\tan\alpha }\left(u\cos\alpha -R\frac{1-\cos\psi }{\cos\psi }\right)-\frac{s/2-u\sin\alpha }{r_{1}}, \end{array}$$

which corresponds to *φ* = (*u*, *ψ*) parameter relationship.

To determine the meshing surface, the expressions (9) (20), and (24) are substituted into the equations in (12). The meshing surface is given by the following formal parameter relationships:

(26) $$\begin{array}{c} X=X\left(u,\psi \right),\\ Y=Y\left(u,\psi \right),\\ Z=Z\left(u,\psi \right). \end{array}$$

Equations of the crowned tooth surface are obtained if (9), (12), (20), and (24) are substituted into (15). They are expressed by the following parameters:

(27) $$\begin{array}{c} x_{1}=x_{1}\left(u,\psi \right),\\ y_{1}=y_{1}\left(u,\psi \right),\\ z_{1}=z_{1}\left(u,\psi \right). \end{array}$$

The axial profile is given by the following parameter relationship for any axial section *z*_1_ = *K* = constant:

(28) $$\begin{array}{c} u=\frac{1}{\cos\alpha }\left(\frac{K}{\sin\psi }-R\right). \end{array}$$

## 5. The Comparison of the Two Models

Two types of mathematical models have been developed for the determination of crowned tooth surface of the coupling hub. Calculations were carried out for formulas of both models by Mathcad. The differences between the results have been determined in case of a gear coupling for a given geometry. It is suitable to compare the two profiles of the two different models in any axial section using arbitrary input data. The comparison is performed by polar coordinates, so that both profiles are determined in polar coordinates, and both polar angles are calculated at a given radius. The deviation is measured as the difference between two polar angles. The difference of these angles Δ*θ* is calculated so that the polar angle of profile-shifted profile is extracted from polar angle of the two-parameter enveloped profile.

Our applied input parameters of the calculations are the following:

 number of teeth *n* = 40,
 module *m* = 3 mm,
 angle of base profile *α* = 20°,
 face width *b* = 20 mm,
 crowning radius *R* = 100 mm,
 profile shifting coefficient in central plane *x*_01_ = 0.

It was experienced that the two profiles coincide with one another in the central plane. Moving away from the central plane, the difference increases, but the order of error remains insignificant. For example, we get Δ*θ* = (−2.15,…, 2) · 10^−4^ degree in the axial section, when the coordinate *z*_1_ = ±2 mm, and Δ*θ* = (−1.35,…, 1.2) · 10^−3^ degree at *z*_1_ = ±5 mm. Generally, negative values are obtained when the radius is less than the radius of pitch circle (*r*_*y*1_ < *r*_1_) and the positive values appear if *r*_*y*1_ > *r*_1_.

**Figure 6:**
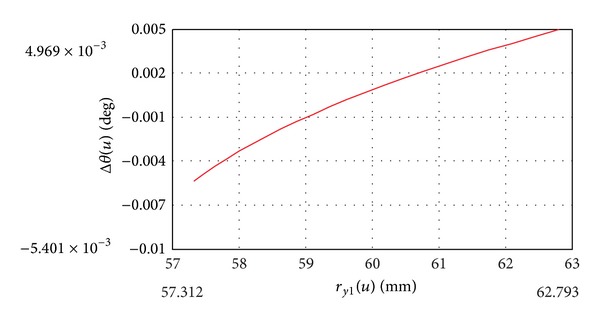
Deviation between two profiles at tooth ends.

Figure 6 shows the angle deviation Δ*θ* as a function of radius *r*_*y*1_ in the axial section *z*_1_ = ±10 mm. It exhibits that the deviation changes from −5.4 · 10^−3^ degree to 5 · 10^−3^ degree approximately.

## 6. Conclusion

On the base of the performed calculations, it was found that both models can be used for further investigation of the gear coupling. According to our calculations, the difference in the profiles for two examined models is very small. Knowing the teeth surfaces of the hub and the sleeve, it is possible to simulate the operation, to analyze the effect of angular misalignment, to investigate the changes in motion transmission, and to calculate the contact stress using the specified curvatures at the contact points.
